# The EU Health Technology Assessment Regulation Halo Effect: Are Cross-Functional Teams Ready?

**DOI:** 10.3390/jmahp13010003

**Published:** 2025-01-30

**Authors:** Sian Tanner, Rebecca Coady, Ana Lisica, Edel Falla, Anke van Engen

**Affiliations:** 1 IQVIA, 1101 CT Amsterdam, The Netherlands; anke.vanengen@iqvia.com; 2 IQVIA, London W2 1AF, UK; rebecca.coady@iqvia.com (R.C.); ana.lisica@iqvia.com (A.L.); edel.falla@iqvia.com (E.F.)

**Keywords:** EU HTA regulation, EU HTA process, innovative health technologies, patient access, European Access Environment, HTA procedures, EU HTA initiative, HTA bodies, European Access Academy, Market Access Society, HTA community

## Abstract

The focus of manufacturers preparing for implementation of the EU HTA Regulation (HTAR) in 2025 has understandably been on their market access teams, and how they can be best equipped to adapt to this significant change. Considering the critical nature of market access in ensuring innovation reaches patients, it should be no surprise that the EU HTAR will have impacts far beyond this function. Here, we utilize published EU HTAR guidance, a pragmatic literature review, internal analysis, and insights from engagements with manufacturers, to outline some of the key cross-functional considerations arising from JSC and JCA, and how manufacturers should account for these in their EU HTAR readiness plans.

## 1. Introduction

The potential for positive impacts from the EU HTAR (HTA Regulation) [1] on market access in Europe has been well documented [2,3,4,5,6]:

- Acceleration and harmonization of patient access across Member States (MSs);
- Opportunity to seek efficient HTA body and regulatory advice on evidence requirements via JSC (Joint Scientific Consultation);
- Eventual reduction in effort required by MSs during local reimbursement evaluations by leveraging the JCA (Joint Clinical Assessment);
- Increased patient representation and involvement in HTA.

It is also clear that concerns remain over uncertainties in the JSC and JCA processes [7]:

- How to plan for and resource the significant evidence generation needs for JCA, earlier than ever before (especially for biostatistics teams);
- How to align regulatory and access strategies and effectively predict the PICO scope (population, intervention, comparator, outcome) [8], especially given the lack of direct PICO information from JSC, insufficient JSC capacity, and lack of manufacturer role in the scoping process;
- How to plan for the early engagement of clinical stakeholders given implications for downstream EU HTAR conflict of interest [9,10,11];
- How to navigate the current uncertainty on triggers for incomplete dossiers, especially acceptable rationales for being unable to address a given PICO [12];
- When clarity will be provided on how local reimbursement and pricing dossiers will evolve [13,14,15];
- Whether provision and uptake of training for Patient Advocacy Groups is sufficient to support their role in JCA.

…to name only a few.

But, while many market access teams have spent significant time and energy preparing for the arrival of the EU HTAR in January 2025, what they may have had less opportunity (or authority) to do is prepare their cross-functional colleagues for impacts extending beyond European market access. We have utilized published EU HTAR guidance, a pragmatic literature review, internal analysis, and insights from engagements with manufacturers, to outline five key themes that access leads should plan for beyond the core JSC and JCA ways of working.

## 2. Global Asset Strategy Implications—It Is a Balancing Act Requiring Senior Leadership Endorsement

There are more perspectives than ever to factor in to asset development decisions: How do you design a clinical trial to generate access-enabling evidence for US, Europe, China, Japan, and beyond? How do you factor in long-term evidence needs for the US post-IRA and for Europe with EU HTAR? When do you need to make prioritization calls if global needs are in conflict? Having a process to weigh these trade-offs and feed into governance decisions is key to avoiding ‘surprise’ late-phase evidence gaps, as well as those arising from manufacturers’ strategic decisions. While this need is not new, there is an increasing level of cross-functionality required at an ever-earlier stage and well-prepared manufacturers are redesigning ways of working to support this.

Call to action: Cross-functional leaders will need to factor EU HTAR requirements and implications into overarching asset strategy (R&D, commercial, regulatory, and access), and evaluate the trade-off between any conflicting needs driven by these policy trends.

## 3. Impacts Beyond the EU—Regional and Local Colleagues Outside of Europe Need to Be Ready

JCA’s direct impact is expected from Q1 2026 with the much earlier, publicly visible English-language HTA report available to be leveraged by HTA and payer bodies globally. Similarly to the way UK NICE reports are referenced (formally or informally) around the world, it is expected that JCA reports will have broad influence; markets such as Canada, Australia, New Zealand and Japan are particularly likely to leverage JCA, provided timings and regulatory labels align. Not only does this potentially impact individual launches, but over time the JCA reports are expected to raise HTA standards globally with greater uptake of this robust methodology. EU HTAR is also likely to continue to inspire further international collaborations for joint HTA decision making, such as the Health Economics Methods Advisory (HEMA) group established by ICER in the US with Canada and the UK [16].

Call to action: Market access and HEOR teams outside of the EU should be familiar with EU HTA methodologies and proactively use the final JCA PICO scope to begin addressing overlap with non-EU evidence requirements, getting a jump-start on global readiness. Global value propositions and objection handlers should be shared early and proactively with non-EU affiliates, so they can leverage upsides or address challenges from the JCA report in payer interactions.

## 4. Launch Sequence Evolution—Commercial Needs to Be in the Loop, and Geographic Priorities May Shift

Political pressure to launch in all EU Member States earlier is likely to increase for manufacturers, delivering on the access equity and acceleration at the heart of the EU HTAR. Although there is uncertainty over how the regulation, in its current form, will achieve this goal, it could be sped up by incentives to adopt JCA/trigger mandatory local reimbursement filings at MS level, such as the EU pharmaceutical legislation reform (e.g., data exclusivity incentives for pan-EU launch within 2 years of marketing authorisation [17]). This would change the traditional norm of staggering EU launches based on commercial priority and could change the attractiveness of the business case of a European launch.

Does Europe’s position in the global launch sequence need to be reconsidered? The downside scenario of EU HTAR is that the burdensome evidence generation requirements, high resource requirements for simultaneous launch in 27 MSs, and the prominence of other markets as commercial priorities (CN, JP, BR, etc.), could further deprioritize Europe in launch planning—particularly for near-term launches during this phase of EU HTAR implementation uncertainty. Manufacturers may evaluate the risks of having the JCA report available early to global priority markets, and delay launch in Europe if the evidence package is likely to be challenged. However, some stakeholders believe a more streamlined and unified EU process will strengthen Europe’s position in the long term. Either way, cross-functional inputs on the clinical development sequence and regulatory strategy are needed given the unavoidable consequences on market access planning.

Call to action: Close collaboration between R&D, regulatory, and market access is needed for the development of launch sequences, both for indication-specific strategies and for lifecycle planning. Changes to launch sequences within the EU will also require the re-evaluation of ways of working, affiliate structure, and resourcing.

## 5. Brand Perception—JCA Will Impact Brand Image and Field Forces Need to Be Trained

**Figure 1:**
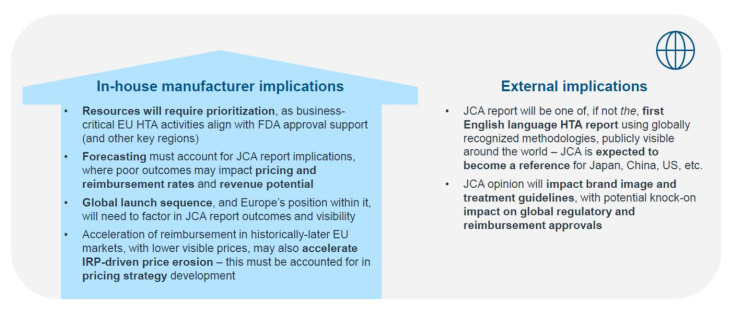
EU HTAR will have global implications for manufacturers, both internally and externally, and must be factored into launch preparations.

Despite the JCA report containing ‘no value judgement’ on the health technologies it assesses, it is unlikely that these reports will not be considered as either favourable or unfavourable upon reading them—based on the tone or assessment of the strength of evidence. These reports will be widely available and have the potential to impact various stakeholders’ perceptions of a brand [see Figure 1]: KOLs and payers will be able to leverage JCA reports to inform their decision making, either casting doubt on the evidence package or suggesting patient subgroups that will receive the most clinical value from an upcoming launch. In the longer term, JCA reports may be leveraged in the development of clinical and payer guidelines. The commercial implications of a more or less favourable JCA report will be particularly key when reports for competitors in the same space are published on a similar time frame. Similarly, the absence of a JCA report at all due to non-compliance discontinuation (e.g., insufficient justification for non-addressed PICOs, or not meeting JCA timelines) is expected to reflect poorly on both the asset and the manufacturer—a perception that is likely to be difficult to shift when it comes to local reimbursement discussions.

Call to action: Commercial teams should scenario plan for potential impacts on brand perception from the JCA report and ensure that in-field teams are familiar with the takeaways. In early development, market access and commercial teams must ensure that their respective strategies are complementary to avoid a JCA dossier strategy that later hampers commercial goals.

## 6. Pricing Impact—Forecasting Teams Need to Be Scenario Planning for JCA

When the EU HTAR eventually achieves its goal of accelerating access equity across MS, there will be consequences for visible prices. We previously explored the impact of International Reference Pricing (IRP) scenarios based on potential changes to EU launch sequences and found that only a few key ‘price taking’ markets need to accelerate to erode average list prices in Europe faster over the first 2 years post launch [18]. Although there could be some individual upside cases; for example, if typically later markets, such as Poland, conclude their assessments faster and reference markets with higher-price markets (e.g., Germany, France) before the smaller, lower-cost markets launch.

Other less direct pricing impacts from EU HTAR could include exposing payers to a broader basket of comparators in the JCA report, potentially providing leverage for them to push for lower priced comparators they may not have otherwise considered, or new subpopulations they can leverage to restrict reimbursement or lower weighted prices. There is some potential for an upside, as manufacturers who do develop a broader evidence base against PICOs with higher priced comparators may have the potential to bring those benchmarks into negotiations. While national list prices may be more resistant to indirect impacts from the JCA, in markets where subnational level payers can demand further confidential discounts there is an uncertainty around how these stakeholders may use the additional information available to them within pricing negotiations.

Call to action: Shifting IRP implications need to be accounted for in long-range forecasting, pricing governance, and even business cases for the commercial potential of future launches. In the longer term, market access teams should monitor for shifts away from traditional payer archetypes towards more markets leveraging JCA to exert downwards pricing pressure and consider the implications for pricing negotiations.

## 7. What Are the Solutions for Manufacturers Preparing for EU HTAR?

Making the most of EU HTAR opportunities while minimizing the risks will require cross-functional best practices in the coming years, including:

- Leadership endorsement for building EU HTAR considerations into early strategic planning and trade-off assessments;
- The efficient use of extensive JCA preparations beyond the EU, empowering all affiliates to engage with the PICO scope and JCA dossier narrative;
- The evaluation of global launch sequences considering evolving R&D, regulatory and market access needs, and the optimal internal processes and resources needed to deliver [see Figure 1];
- Readying commercial teams for the brand perception impact of JCA, and factoring this into strategic scenario planning and field force training;
- Monitoring for changes in international reference pricing and payer approaches to pricing that may influence forecasting and asset business cases.

Of course, there are still actions for market access teams aside from preparing for JSC and JCA itself: for those with assets in-scope for the first phase, the monitoring of in-scope EMA filings and early JCA reports (Q4 2025+), the development of KPIs for EU HTAR success (internally and externally), after-action reviews for early JSC/JCA assets, peer benchmarking, and the evaluation and optimization of ways of working. For manufacturers still embarking on their EU HTAR readiness journey (e.g., where assets are not in scope until 2028 onwards), there is time to implement organizational changes to support the cross-functional considerations outlined here through JSC/JCA process design, pilot PICO simulations/dossier practice, cross-functional team training, earlier integrated HTA-standard evidence planning, and asset-specific JCA strategy planning.
